# Impact of Intercostal Artery Reinsertion on Neurological Outcome after Thoracoabdominal Aortic Replacement: A 25-Year Single-Center Experience

**DOI:** 10.3390/jcm13030832

**Published:** 2024-01-31

**Authors:** Florian Helms, Reza Poyanmehr, Heike Krüger, Bastian Schmack, Alexander Weymann, Aron-Frederik Popov, Arjang Ruhparwar, Andreas Martens, Ruslan Natanov

**Affiliations:** 1 Division for Cardiothoracic-, Transplantation- and Vascular Surgery, Hannover Medical School, Carl-Neuberg-Str. 1, 30625 Hannover, Germany; 2 Clinic for Cardiac Surgery, University Clinic Oldenburg, 26133 Oldenburg, Germany

**Keywords:** aortic surgery, thoracoabdominal aorta, intercostal artery reinsertion, spinal cord protection

## Abstract

Background: Intercostal artery reinsertion (ICAR) during thoracoabdominal aortic replacement remains controversial. While some groups recommend the reinsertion of as many arteries as possible, others consider the sacrifice of multiple intercostals practicable. This study investigates the impact of intercostal artery reinsertion or sacrifice on neurological outcomes and long-term survival after thoracoabdominal aortic repair. Methods: A total of 349 consecutive patients undergoing thoracoabdominal aortic replacement at our institution between 1996 and 2021 were analyzed in a retrospective single-center study. ICAR was performed in 213 patients, while all intercostal arteries were ligated and sacrificed in the remaining cases. The neurological outcome was analyzed regarding temporary and permanent paraplegia or paraparesis. Results: No statistically significant differences were observed between the ICAR and non ICAR groups regarding the cumulative endpoint of transient and permanent spinal cord-related complications (12.2% vs. 11.8%, *p* = 0.9). Operation, bypass, and cross-clamp times were significantly longer in the ICAR group. Likewise, prolonged mechanical ventilation was more often necessary in the ICAR group (26.4% vs. 16.9%, *p* = 0.03). Overall long-term survival was similar in both groups in the Kaplan–Meier analysis. Conclusion: Omitting ICAR during thoracoabdominal aortic replacement may reduce operation and cross-clamp times and thus minimize the duration of intraoperative spinal cord hypoperfusion.

## 1. Introduction

Neurological complications after open surgical thoracoabdominal aortic repair are relatively common and can be very disabling. Large patient series have shown an incidence of 5.1% for permanent paraplegia or paraparesis [1]. Within this population, the highest rates of adverse spinal cord-related events were observed in patients with Crawford type III disease, with an incidence of 7.5%. To reduce the risk of spinal cord injury, cerebrospinal fluid drainage, deep hypothermia, optimized patient blood management, and distal aortic perfusion during cardiopulmonary bypass have been proposed as protective measures [2,3,4]. Furthermore, despite inconsistent evidence, intercostal artery reinsertion (ICAR) is still today considered an integral part of spinal cord protection strategies in many centers [5,6,7]. Although a significant reduction in neurologic complications has been achieved using a combination of these strategies, the isolated role of ICAR remains unclear. 

The rationale for the majority of neuroprotective strategies is based on the so-called collateral network concept [8]. According to this framework, which is based on both experimental and clinical observations, three factors need to be considered for spinal cord perfusion: First, the existence of an axial vascular network within the spinal canal and paravertebral tissues needs to be taken into account. This network includes both horizontal and vertical collaterals and thus facilitates trans-segmental perfusion. Second, not only the intercostal arteries but also the vertebral arteries arising from the subclavian arteries and the hypogastric arteries are relevant for the perfusion of this collateral network. Finally, the collateral network is considered an adaptive structure that facilitates compensation for the loss of an inflow to the spinal cord perfusion by increasing the formation of collateral connections to alternative inflows.

Considering this concept and with increasing clinical experience, ICAR during thoracoabdominal replacement has become increasingly controversial over the last decade. While some groups recommend the reinsertion of as many arteries as possible [9,10,11], others consider the sacrifice of multiple intercostals practicable [7,12,13]. This study investigates the impact of ICAR on neurological outcomes and long-term survival after open thoracoabdominal repair.

## 2. Patients and Methods

### 2.1. Patients

Between January 1996 and June 2021, a total of 349 consecutive patients underwent open thoracoabdominal aortic repair at our tertiary center. Patient characteristics, intraoperative parameters, and postoperative complications were collected continuously in our institutional database and analyzed retrospectively for this study. If a missing value was discovered during the retrospective evaluation, it was added with the help of the clinic’s internal documentation and archiving so that completeness of the data was achieved for the parameters used.

### 2.2. Study Design and Variables

The study population was divided into two groups: ICAR patients with ICAR and a non ICAR patient population without intercostal reinsertion. Spinal cord-related neurological complications were defined as temporary and permanent paraparesis or paraplegia and analyzed separately as well as combined as a composite endpoint. Paraplegia and paraparesis were diagnosed as clinical diagnoses during the physical examination. If the routine examination revealed a suspicious finding for paraplegia or paraparesis, this diagnosis was verified by a neurologist’s consultation. If the distribution of deficits detected in the clinical examination remained inconclusive with regard to a potential ischemic etiology, further diagnostic imaging in the form of a cranial and/or spinal CT was performed to rule out potential differential diagnoses such as paraplegia or paraparesis of cerebral origin. The spinal cord-associated neurological deficits were considered transient if they had regressed by the time of discharge and were otherwise defined as permanent. 

The extent of repair was classified following the Crawford classification of thoracoabdominal diseases [14]. Acute kidney failure was defined as a three-fold increase in serum creatinine or a urine output of less than 0.5 mL per kg body weight per hour for 24 h [15]. Low cardiac output syndrome was defined as a cardiac index < 2.2 L/min × BSA (body surface area in m^2^) or dependency on catecholamine therapy. The necessity of re-intubation or non-invasive ventilation after previous spontaneous breathing was defined as respiratory failure.

### 2.3. Preoperative Assessment

CT angiography was used as the gold standard technique for preoperative diagnostics and planning of the operation. Additionally, electrocardiography, spirometry, and transthoracic echocardiography, with a particular focus on the left ventricular function and the aortic valve function and morphology, were performed as the standard of care prior to every non-emergent thoracoabdominal aortic repair operation. Further, preoperative diagnostics such as left heart catheterization, transesophageal echocardiography, or duplex sonography of the carotid arteries were performed based on the individual patient characteristics and risk factors. If a patient had a contraindication for CT angiography, magnetic resonance angiography was used as an alternative imaging technique. Preoperative diagnostics were primarily directed at assessing the operability, the exact morphology, and the extent of the aortic pathology, and the necessity and possibility of reinsertion of the supra-aortic, visceral, and lower limb vessels. While larger intercostal arteries could be visualized by CT angiography, no further specific imaging techniques for the visualization of single intercostal arteries, such as the Adamkiewicz-artery, were performed.

### 2.4. Surgical Technique

As standard access for thoracoabdominal aortic replacement, a left-sided thoracotomy in the 5th to 7th intercostal space, starting caudal to the angulus inferior of the scapula and expanding into a paramedian abdominal incision, was used. The abdominal aorta was accessed through a retroperitoneal approach. The diaphragm was incised, and an Omni-tract^®^ (Integra LifeSciences, Princeton, NJ, USA) retractor was placed for exposure. Heparinization was based on body weight with a dose of 400–500 IU/kgBWT (body weight). The activated clotting time (ACT) was measured prior to initialization of cardiopulmonary bypass, periodically during bypass time, and after antagonization with protamine. The target ACT during cardiopulmonary bypass was >450 s. Following systemic heparinization, cardiopulmonary bypass was initiated via the femoral vessels, and cooling was started. After proximal and distal clamping, proximal and distal anastomosis was performed, and hemostasis was obtained. If ICAR was performed, intercostals were excised from the native aortic wall as buttons and connected to the aortic prosthesis by direct anastomoses. Otherwise, sacrificed intercostal arteries were ligated to avoid back-bleeding. If necessary, visceral and renal arteries were reinserted using additional vascular grafts or prefabricated branches of the prostheses as applicable. After rewarming and hemostasis, graft inclusion with native aortic tissue was performed, and the wound was closed in a standard fashion. The patients were then transferred to our cardiac surgical intensive care unit for further stabilization. In vivo, optical spectroscopy monitoring was implemented as a standard monitoring technique in extensive cases, including the distal aortic arch and proximal descending aorta, from 2018 onwards.

### 2.5. Statistical Analysis

Statistical analysis was performed using IBM SPSS Statistics 28 (IBM Corp., Armonk, NY, USA, 1989, 2021). Continuous variables were tested for normal distribution using the Kolmogorov–Smirnov test. Normally, distributed data are given as the mean ± standard deviation (SD). For non-normally distributed data, median and interquartile ranges (Q1–Q3) are given. Homoscedasticity was tested using the Lavene test, and continuous variables were compared using the *t*-test or the Mann–Whitney test. Categorial variables are given as total numbers (*n*) and percentages. Differences were considered significant at a *p* < 0.05. An inverse probability-weighted generalized model was used for risk factor analysis for the composite endpoint composed of temporary and permanent paraparesis and paraplegia. For this, continuous variables were converted into binary variables with the cut-off value above 3. For this, continuous variables were converted into binary variables with the cut-off value at the third quartile for the age at operation and operation-, bypass-, and cross-clamp times. Inverse probability weighting was performed for Crawford’s extents I–V to correct for potential bias due to the unequal frequency of the Crawford types in each group. The full list of the variables that were analyzed for the model is provided in Supplementary Table S2. The Kaplan–Meier survival estimates, including the log-rank test, were used to analyze survival.

## 3. Results

**Figure 1:**
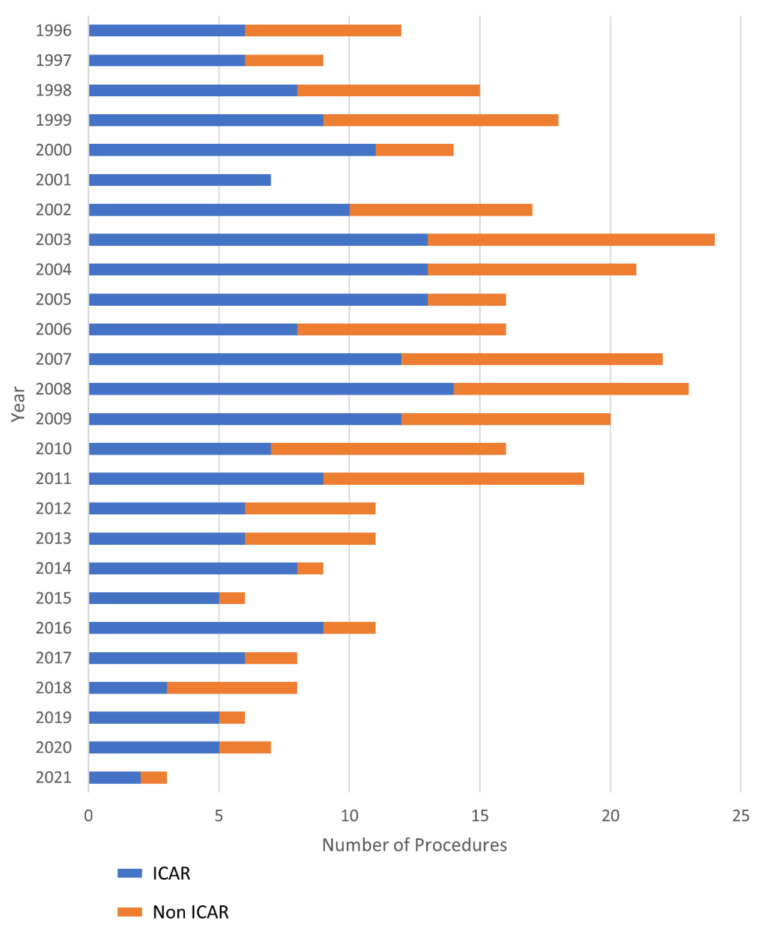
Absolute numbers of thoracoabdominal aortic replacements with (blue) or without (orange) intercostal artery reinsertion performed at our institution from 1996 to June 2021.

ICAR was performed in 213 (61%) out of 349 cases. Throughout the study period, minor fluctuations in the annual reinsertion rate were observed, ranging from 53.5% to 77.7% within the interquartile range. No discernible consistent trend towards an increase or decrease in reinsertions over time was identified, as depicted in Figure 1. The median patient age was significantly higher in the non ICAR-group (64 years, IQR 54–71 vs. 62 years, IQR 52.5–68, *p* = 0.04), while the median body mass index was significantly higher in patients of the ICAR group (26.2, IQR 23.4–28.7 vs. 24.7, IQR 22.3–27.4, *p* = 0.005) (Table 1). The urgency of the operation was significantly higher in patients in which all intercostal arteries were sacrificed, with 13.2% vs. 5.6% of cases performed as emergency operations (*p* = 0.013, Table 2). Crawford’s extent differed between the groups for type II and type IV. While Crawford type II was more frequently present in the ICAR group, the prevalence of Crawford type IV was higher in the non ICAR group (Table 3).

Operation, bypass, and cross-clamp times were significantly longer when ICAR was performed (Table 4). For spinal cord-related neurological outcomes, no statistically significant differences were observed. The cumulative endpoint of permanent and temporary paraplegia or paraparesis occurred in 12.2% of the ICAR group and 11.8% of cases in the non ICAR patients (*p* = 0.9). Likewise, no significant differences were observed in the separate analysis of the individual neurological outcome parameters (Table 5) or the separate assessment of each Crawford extent (Supplementary Table S1).

**Figure 2:**
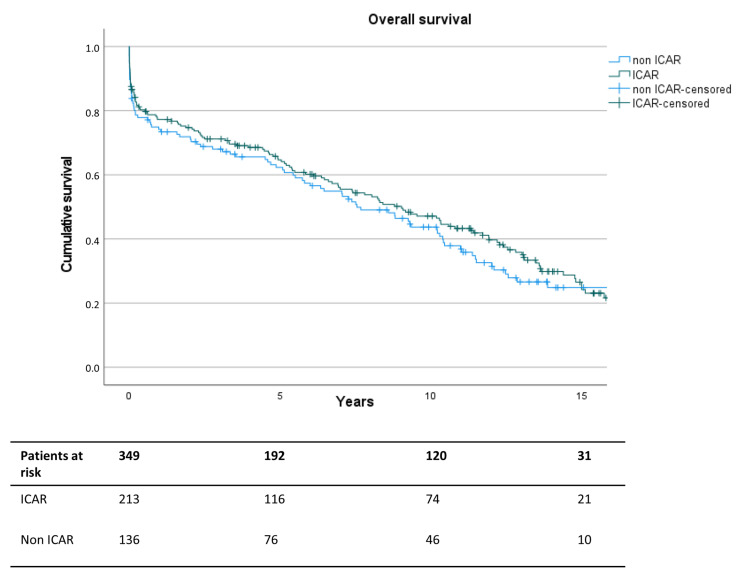
Kaplan–Meier analysis for overall mortality after thoracoabdominal aortic replacement with (dark green) or without (blue) intercostal artery reinsertion.

In the early postoperative course, more respiratory complications in the form of prolonged ventilation times, respiratory failure, and the need for tracheostomy were observed in the ICAR patients (Table 6). Likewise, the postoperative hospital stay was significantly longer when ICAR was performed (15 days, IQR 11–22 vs. 13 days, IQR 9–20, *p* = 0.03). Long-term survival showed no significant differences between both groups (*p* = 0.393, Figure 2). Similarly, the subgroup analysis for each Crawford type showed no significant differences between the ICAR and non ICAR groups.

The inverse probability-weighted generalized linear model revealed bypass and cross-clamp times, as well as diabetes comorbidity, as relevant risk factors for the cumulative spinal cord-related neurological complication endpoint. ICAR and the Crawford II extent of repair did not contribute significantly to the multivariate risk model (Table 7).

## 4. Discussion

The main findings of this study can be summarized as follows: (1) ICAR in thoracoabdominal aortic replacement seemed not to reduce the overall incidence of spinal cord-related adverse neurological events. (2) Operation times were significantly longer when ICAR was performed. (3) In the multivariate analysis, bypass and cross-clamp times and diabetes, but not intercostal artery sacrifice, were identified as possible relevant risk factors for spinal cord-related adverse events.

ICAR remains one of the most frequently used strategies for spinal cord protection during and after thoracoabdominal aorta replacement. In the early days of thoracoabdominal repair, Svensson and Coselli reported an up to three-fold higher incidence of adverse neurological events if intercostal arteries were sacrificed compared to extensive reimplantation of intercostal and lumbar arteries, especially in the region of the thoracoabdominal junction [16]. Consequently, they recommended the reinsertion of as many intercostal and lumbar arteries as possible, particularly between TH11 and L1. Following this evidence and recommendation, liberal ICAR was initially implemented as an integral factor for spinal cord protection in many centers and often remained unchanged until today [6,7,17,18]. Further studies supported these findings and especially stressed the importance of reinserting the Arteria radicularis magna or Adamkiewicz-artery located at TH12 or L1. Here, Ogino et al. reported a spinal cord injury rate of only 1.1% using magnetic resonance imaging (MRI) for preoperative visualization and targeted reinsertion of the Adamkiewicz- artery and further perfused intercostals in combination with intraoperative motor-evoked potential monitoring [19]. Following this, Acher et al. showed that adding ICAR to neuroprotective strategies could further reduce the risk of spinal cord injury [9].

Contrary to that, Etz et al. reported a series of one hundred patients undergoing replacement of the thoracoabdominal aorta, in which extensive intercostal artery sacrifice of an average of eight segmental pairs was performed [13]. Despite this, the rate of permanent paraplegia was only 2%, although intercostals in the Adamkiewicz-region TH7-L1 were also liberally ligated. This observation was later supported by Wynn et al., who showed no statistically significant reduction in spinal cord injury rates when ICAR was performed compared to a historical control group without ICAR [12].

In our single-center experience presented here, ICAR did not seem to be associated with lower rates of paraplegia and paraparesis when considering all Crawford extents cumulatively. When ICAR was performed, operation times were longer, and postoperative respiratory complications were more frequent. Multivariate analysis revealed that bypass and cross-clamp times were significant risk factors for adverse spinal cord-related events. Thus, the prolongation of the operation time in the setting of intercostal reinsertion may have canceled out the potential benefits of reinsertion. Additionally, diabetes comorbidity was found to be a significant risk factor for spinal cord-related adverse neurological events after open thoracoabdominal aortic replacement. With regard to the underlying mechanism, an impaired collateralization of the spinal cord in the context of diabetic microangiopathy could be assumed. This could have reduced the resilience of the spinal cord to malperfusion and thus led to higher paraparesis and paraplegia rates.

In the synoptic review of the findings, our experience further supports the collateral network concept first introduced by Griepp et al. in 2007 [8]. Following this concept, spinal cord perfusion relies on a collateral vascular network fed not only by segmental intercostal and lumbar arteries but also by subclavian, pelvic, and hypogastric branches. Consequently, strategies for spinal cord protection during thoracoabdominal aorta replacement can target either transient intraoperative hypoperfusion or long-term spinal cord injury due to sustained malperfusion of the collateral network. For a reduction in intraoperative malperfusion, numerous approaches to minimize ischemic spinal cord damage during the operation itself have been introduced and have been proven effective over the last decades. Among those, lower body perfusion [20], hypothermia [4], and cerebrospinal fluid drainage [21] have been proven to be the most relevant. In our clinical practice, we applied all of these strategies in combination with ICAR when suitable. This constitutes a limitation of the retrospective study presented here, as these additional strategies were used in parallel with intercostal artery reinsertion in the interests of patient safety and good clinical practice. 

## 5. Limitations

As this study is designed as a retrospective single-center study, limitations, including a possible selection bias, need to be considered. In addition, differences in the subgroup analysis may not have been recorded due to the partly small number of cases. Since paraplegia and paraparesis were diagnosed by clinical examination, a potential confounding effect of death occurring before the clinical diagnosis of paraplegia and paraparesis was made in cases of early in-hospital mortality cannot be precluded. Patency rates of the left subclavian artery and the hypogastric artery have not been investigated systematically in our database. Continuous innovations in endovascular techniques may have also influenced the patient selection over the study period. While no clear trends in the decision for or against intercostal artery reinsertion or with regard to the surgical technique for intercostal reinsertion were observed over time, slight differences in operating times and individual technical details between different surgeons are noticeable, which can be considered a potential limitation to this study.

## 6. Conclusions

With the intent of ensuring long-term segmental spinal cord protection, ICAR was proposed by many authors. However, in this study, we showed that prolonged bypass and cross-clamp times were associated with spinal cord injury, while ICAR did not seem to significantly reduce the adverse neurological event rates. This implies that longer intraoperative spinal cord hypoperfusion appears to be more relevant for the neurological outcome than a potential long-term impairment of segmental spinal cord perfusion by intercostal artery sacrifice.

Overall, the results of this study suggest that the primary focus of strategies to protect the spinal cord in the thoracoabdominal aortic repair should be on limiting intraoperative spinal cord hypoperfusion, particularly by minimizing the operative time, rather than dwelling on extensive reinsertion of intercostal arteries. With endovascular techniques becoming increasingly popular, the role of the intercostal arteries in the spinal cord remains of utmost importance for the future. In this regard, our results suggest that intercostal artery sacrifice appears to be justifiable in terms of the spinal cord-associated neurologic outcome. This also potentially paves the way for extensive endovascular aortic repair where intercostal artery reinsertion is not feasible. Nonetheless, these results can only provide an initial indicator and cannot be readily extrapolated to endovascular techniques. Thus, further studies evaluating the spinal cord-associated neurological outcome in extensive endovascular aortic repair are needed to answer this question in the future.

## Figures and Tables

**Table 1:** Preoperative characteristics.

Characteristics	ICAR Group	Non ICAR Group	*p*-Value
Sex (male)	152 (71.4%)	86 (63.2%)	0.112
BMI (kg/m^2^)	26.20 (23.41–28.72)	24.73 (22.30–27.44)	0.005
Age at operation (years)	62 (52.5–68)	64 (54–71)	0.042
Marfan	27 (12.7%)	13 (9.6%)	0.373
Hypertension	127 (59.6%)	83 (61%)	0.794
Hyperlipidemia	30 (14.1%)	31 (22.8%)	0.037
Diabetes	11 (5.2%)	9 (6.6%)	0.569
Coronary artery disease	60 (28.2)	41 (30.1%)	0.691
Cerebrovascular disease	11 (5.2%)	14 (10.3%)	0.070
Chronic renal disease	38 (17.8%)	28 (20.6%)	0.523
COPD	28 (13.1%)	17 (12.5%)	0.861
Tabacco smoking	39 (18.3%)	22 (16.2%)	0.609
Peripheral vascular disease	27 (12.7%)	25 (18.4%)	0.144
Re-do (prior cardiac)	85 (39.9%)	51 (37.5%)	0.653
Re-do (prior open aortic)	115 (54%)	65 (47%)	0.259
Prior EVAR	2 (0.9%)	1 (0.7%)	0.841
Prior TEVAR	2 (0.9%)	3 (2.2%)	0.331

BMI = body mass index (kg/m^2^), COPD = chronic obstructive pulmonary disease, EVAR = (abdominal) endovascular aortic repair, TEVAR = thoracic endovascular aortic repair.

**Table 2:** Urgency.

Characteristics	ICAR Group	Non ICAR Group	*p*-Value
Elective	189 (88.7%)	107 (78.7%)	0.011
Urgent	11 (5.2%)	9 (6.6%)	0.569
Emergent	12 (5.6%)	18 (13.2%)	0.013

**Table 3:** Extent of repair.

Crawford Extent of Repair	ICAR Group	Non ICAR Group	*p*-Value
I	37 (17.4%)	16 (11.8%)	0.155
II	54 (25.4%)	10 (7.4%)	<0.001
III	82 (28.5%)	41 (30.1%)	0.111
IV	8 (3.8%)	45 (33.1%)	<0.001
V	30 (14.1%)	15 (11.0%)	0.406

**Table 4:** Intraoperative characteristics.

Characteristics	ICAR Group	Non ICAR Group	*p*-Value
FET-completion	9 (4.2%)	2 (1.5%)	0.151
Selective renal artery perfusion	107 (50.2%)	70 (51.5%)	0.822
CSF-drainage	43 (20.2%)	26 (19.1%)	0.807
Left-heart-bypass	1 (0.5%)	3 (2.5%)	0.137
Circulatory arrest	31 (14.6%)	14 (10.3%)	0.247
Operation time (min)	369.57 ± 113.89	303.77 ± 99.77	<0.001
Bypass time (min)	168.73 ± 75.59	117.70 ± 68.54	<0.001
Cross-clamp time (min)	103 (82–147)	82 (62–129)	<0.001
Intraoperative mortality	4 (1.9%)	3 (2.2%)	0.831

FET = Frozen elephant trunk prosthesis, CSF = cerebrospinal fluid.

**Table 5:** Spinal cord-related neurological outcome.

Characteristics	ICAR Group	Non ICAR Group	*p*-Value
Cumulative spinal cord-related complications	26 (12.2%)	16 (11.8%)	0.902
Stroke	6 (2.8%)	3 (2.2%)	0.725
Temporary paraplegia	3(1.4%)	3 (2.2%)	0.576
Permanent paraplegia	12 (5.6%)	8 (5.9%)	0.922
Temporary paraparesis	3 (1.4%)	1(0.7%)	0.565
Permanent paraparesis	9 (4.2%)	4 (2.9%)	0.537

**Table 6:** Postoperative characteristics.

Characteristics	IC Reinsertion	Non IC Reinsertion	*p*-Value
Ventilation > 72 h	57 (26.4%)	23 (16.9%)	0.033
Respiratory failure	72 (33.8%)	32 (23.5%)	0.041
Tracheostomy	39 (18.3%)	10 (7.4%)	0.004
ARDS	2 (1.8%)	1 (0.7%)	0.841
Pneumonia	15 (7.0%)	6 (4.4%)	0.314
Pulmonary embolism	3 (1.4%)	1 (0.7%)	0.565
Left vocal cord paralysis	9 (4.2%)	2 (1.5%)	0.151
Reanimation	5 (2.3%)	4 (2.9%)	0.733
Sepsis	11 (5.2%)	5 (3.7%)	0.517
Wound infection	17 (8.0%)	5 (3.7%)	0.107
Bleeding requiring re-thoracotomy	29 (13.6%)	17 (12.5%)	0.764
Acute kidney failure	41 (19.2%)	20 (14.7%)	0.276
Dialysis (temporary)	17 (8.0%)	5 (3.7%)	0.107
Dialysis (permanent)	15 (7.0%)	10 (7.4%)	0.913
Atrial fibrillation	3 (4.9%)	5 (3.7%)	0.167
Myocardial infarction	4 (1.9%)	2 (1.5%)	0.775
Cardiac tamponade	2 (0.9%)	0 (0.0%)	0.257
LCOS	7 (3.3%)	8 (5.9%)	0.244
ECMO	6 (2.8%)	1 (0.7%)	0.176
GI ischemia	6 (2.8%)	3 (2.2%)	0.725
GI bleeding	5 (2.3%)	4 (2.9%)	0.733
GI obstruction	3 (1.4%)	4 (2.9%)	0.319
Pancreatitis	4 (1.9%)	2 (1.5%)	0.775
ICU stay (d)	4 (2–11)	3 (2–6)	0.066
Ventilation time (h)	22.78 (13.87–83.78)	19.18 (12.14–48.07)	0.042
Hospital stay (d)	15 (11–22)	13 (9–20)	0.027
In-hospital mortality	39 (18.3%)	28 (20.6%)	0.598
30-day mortality	28 (13.1%)	22 (16.2%)	0.431

ARDS = acute respiratory distress syndrome.

**Table 7:** Significant factors for spinal cord-related complications in the inverse probability-weighted generalized linear model.

Characteristics	Odds Ratio	95% CI	*p*-Value
Bypass time (>3.Quartile)	1.205	1.049–1.860	0.030
Cross-clamp time (>3.Quartile)	5.846	1.416–24.136	0.015
Diabetes	6.603	1.319–27.873	0.021

## Data Availability

Data used in this study are available from the corresponding author upon request.

## Supplementary Materials

The following supporting information can be downloaded at: https://www.mdpi.com/article/10.3390/jcm13030832/s1, Table S1. Subgroup analysis of the incidence of the cumulative neurological endpoint. Table S2. Factors evaluated for the inverse probability-weighted generalized linear model.
