# Supplementary material for: Impact of Intercostal Artery Reinsertion on Neurological Outcome after Thoracoabdominal Aortic Replacement: A 25-Year Single-Center Experience
Source: J Clin Med. 2024 Jan 31;13(3):832. doi: 10.3390/jcm13030832 (PMC10856124; doi:10.3390/jcm13030832)
Supplement: Supplementary file 1 [file jcm-13-00832-s001.zip › jcm-2832205-supplementary.pdf]

Table S1 – subgroup analysis of the incidence of the cumulative neurological endpoint

Crawford Classification	ICAR group	Non ICAR group	P-Value
**Type I**	3 (8.1%)	2 (12.5%)	0.616
**Type II**	8 (14.8%)	2 (20.0%)	0.678
**Type III**	13 (15.9%)	4 (9.8%)	0.356
**Type IV**	7 (15.6%)	0 (0%)	0.231
**Type V**	2 (6.7%)	1 (6.7%)	1

Table S2 – factors evaluated for the inverse probability weighted generalized linear model

**Age at operation (>3.Quartile)**
**ICAR**
**Sex**
**Marfan**
**Hypertension**
**Hyperlipidemia**
**Diabetes**
**Coronary artery disease**
**Cerebrovascular disease**
**Chronic renal disease**
**COPD**
**Tabacco smoking**
**Peripheral vascular disease**
**Re-do (prior cardiac)**
**Re-do (prior open aortic)**
**Prior EVAR**
**Prior TEVAR**
**Crawford extent II**
**Selective Renal artery perfusion**
**CSF drainage**
**Left heart bypass**<br>**Circulatory arrest**
**Operation time (>3. Quartile)**
**Bypass time (>3. Quartile)**
**Cross-clamp time (>3. Quartile**
